# An N-Terminal Acidic Patch Underlies Differential Inhibition of mRNA Nuclear Export by Human Coronavirus Nsp1 Proteins

**DOI:** 10.3390/v18101157

**Published:** 2026-10-18

**Authors:** Fei Wang, Baoyin Su, Ziyan Huang, Ziyun Cheng, Chunhui Chang, Yunjiao Zhang, Leer Shen, Ziyue Li, Jing Zhang, Xiaohua Chen, Jiaming Lan, Yaming Jiu, Xun Chen, Juan Li, Chengyuan Wang, Ke Zhang

**Affiliations:** 1 Ruijin Hospital, Shanghai Jiao Tong University School of Medicine, Shanghai 200001, China; 2 Shanghai Institute of Materia Medica, Chinese Academy of Sciences, Shanghai 201203, China; bysu@siii.cas.cn (B.S.); huangziyan@simm.ac.cn (Z.H.); chchang@siii.cas.cn (C.C.); jzhang1@simm.ac.cn (J.Z.);; 3 University of Chinese Academy of Sciences, Beijing 100049, China; 4 Department of Infectious Diseases, Zhongshan Hospital, Fudan University, Shanghai 200437, China; 24111210030@m.fudan.edu.cn; 5 Department of Respiratory and Critical Care Medicine, Shanghai Sixth People’s Hospital Affiliated to Shanghai Jiao Tong University School of Medicine, Shanghai 200233, China; zhangyjiao7w@hotmail.com; 6 Department of Infectious Diseases, Shanghai Sixth People’s Hospital Affiliated to Shanghai Jiao Tong University School of Medicine, Shanghai 200233, China; shenleer1998@163.com (L.S.);; 7 Shanghai Institute of Virology, Shanghai Jiao Tong University School of Medicine, Shanghai 200001, China

**Keywords:** Human Coronavirus, Nsp1, mRNA nuclear export, NXF1

## Abstract

Severe acute respiratory syndrome coronavirus 2 (SARS-CoV-2) and related human coronaviruses (hCoVs) regulate host gene expression to facilitate viral replication and evade innate immunity. SARS-CoV-2 nonstructural protein 1 (Nsp1) suppresses host gene expression in part by inhibiting cellular mRNA nuclear export through targeting the mRNA export factor NXF1. However, whether this mechanism is conserved among Nsp1 proteins from diverse hCoVs remains unknown. Here, we demonstrate that Nsp1 proteins from SARS-CoV, SARS-CoV-2, and MERS-CoV, but not those from endemic hCoVs, induce pronounced nuclear accumulation of host poly(A) RNA without altering global transcript levels. Nsp1 proteins from these highly pathogenic hCoVs directly interact with NXF1, whereas endemic hCoV Nsp1 proteins show little or no detectable NXF1 interaction. Structural modeling and mutagenesis identify a conserved N-terminal acidic patch in Nsp1 that contacts the basic surface of the NXF1 RNA recognition motif. Disruption of this interface abolishes the Nsp1-NXF1 interaction and restores host mRNA export while preserving NXF1 interactions with core mRNA export factors. Together, our findings define a conserved structural mechanism by which highly pathogenic hCoV Nsp1 targets NXF1 to inhibit host mRNA export and provide a potential framework for broad-spectrum antiviral intervention.

## 1. Introduction

Outbreaks of highly pathogenic coronaviruses, including Severe Acute Respiratory Syndrome Coronavirus (SARS-CoV), Middle East Respiratory Syndrome Coronavirus (MERS-CoV), and SARS-CoV-2, have caused devastating global public health crises and severe economic disruption [1,2,3]. Beyond these epidemic strains, four endemic human coronaviruses (hCoVs), namely HCoV-OC43, HCoV-HKU1, HCoV-NL63, and HCoV-229E, persistently circulate worldwide, causing mild to severe respiratory illnesses in vulnerable populations [4,5]. The ongoing emergence of novel viral variants and the threat of zoonotic spillovers highlight the urgent need to elucidate fundamental mechanisms of coronavirus–host interactions, thereby uncovering conserved molecular targets for broad-spectrum therapeutic development [6,7].

Coronaviruses possess exceptionally large positive-sense single-stranded RNA genomes that encode up to 16 nonstructural proteins (Nsp1 to Nsp16) within Open Reading Frame 1a/1ab (ORF1a/1ab), which profoundly remodel host gene expression to promote replication [2,8]. Among these, Nsp1 is the first protein expressed upon viral entry and functions as a master regulator of host gene expression shutoff [9,10]. In SARS-CoV and SARS-CoV-2, Nsp1 uses its C-terminal domain to insert into the entry channel of the 40S ribosomal subunit, physically blocking the entry of host mRNA and stalling translation initiation [11,12]. Simultaneously, Nsp1 promotes endonucleolytic cleavage and degradation of host mRNAs while sparing viral transcripts containing specific 5′-end leader sequences [9,13].

In eukaryotic cells, nucleocytoplasmic transport serves as a critical regulatory gateway for gene expression [14,15]. The nuclear export of mature messenger ribonucleoprotein complexes (mRNPs) is primarily mediated by Nuclear RNA Export Factor 1 (NXF1, also known as TAP) in heterodimeric association with NTF2-related export protein 1 (NXT1/p15) [16,17]. NXF1-NXT1 is recruited to mRNPs through export adaptors such as the TREX/THO complex and Aly/REF and facilitates their translocation through the nuclear pore complex (NPC) [18,19]. Rapid export of host transcripts is particularly vital during antiviral responses, as genes encoding type I/III interferons (IFNs) and proinflammatory cytokines must be rapidly transcribed, exported, and translated to establish an antiviral state [20,21]. Consequently, viral pathogens have evolved diverse mechanisms to intercept nucleocytoplasmic export; for instance, influenza A virus NS1 interacts with NXF1 [22,23,24], while SARS-CoV-2 ORF6 targets the NUP98–RAE1 complex to block nuclear import and export machinery [25,26]. Although SARS-CoV-2 Nsp1 has been implicated in suppressing host macromolecular export [27,28,29], whether inhibition of host mRNA nuclear export is conserved across the Alphacoronavirus and Betacoronavirus genera remains unclear. The molecular determinants and structural basis underlying this conserved activity also remain poorly defined. In this study, we systematically compared Nsp1 proteins from seven human coronaviruses representing highly pathogenic and endemic hCoVs. We found that Nsp1 from SARS-CoV, SARS-CoV-2, and MERS-CoV, but not endemic hCoVs, potently inhibits host mRNA nuclear export and interacts with the mRNA export receptor NXF1. Notably, the highly conserved N-terminal acidic patch critical for Nsp1-NXF1 binding and inhibition of host mRNA export is preserved in Nsp1 proteins from highly pathogenic hCoVs. These findings establish a conserved NXF1-targeting mechanism underlying host mRNA export inhibition by highly pathogenic hCoV Nsp1 proteins.

## 2. Materials and Methods

### 2.1. Reagents

Glutathione Sepharose 4B (Cytiva, Marlborough, MA, USA, 17-0756-01), Anti-Flag M2 Magnetic Beads (Sigma-Aldrich, St. Louis, MO, USA, M8823), Ni-NTA Agarose (Smart-Lifesciences, Changzhou, Jiangsu, China, SA004005), Protein A/G agarose (Santa Cruz Biotechnology, Dallas, TX, USA, sc-2003), 3×Flag peptide (GLPBIO, Montclair, CA, USA, GP10149), RNase A (Vazyme, Nanjing, Jiangsu, China, DE111-01), RiboLock RNase (Thermo Fisher Scientific, Waltham, MA, USA, EO0384), TRIzol reagent (Thermo Fisher Scientific, 15596018), TransScript One-Step gDNA Removal and cDNA Synthesis SuperMix (TransGen Biotech, Beijing, China, AT311-02). PerfectStart Green qPCR SuperMix (TransGen Biotech, AQ601-01-V2), Lipofectamine 3000 (Thermo Fisher Scientific, L3000015), jetOPTIMUS transfection reagent (Polyplus, Illkirch-Graffenstaden, France, 117-07), and Hoechst 33258 (Thermo Fisher Scientific, H3569) were used. Oligo(dT)-CY5 probe for RNA-FISH was synthesized by Thermo Fisher Scientific.

### 2.2. Antibodies

The following primary antibodies were used: mouse anti-Flag M2 (Sigma-Aldrich, F1804; 1:2000 for WB, 1:1000 for IF and PLA), rabbit anti-Aly/REF (ABclonal, Wuhan, Hubei, China, A6010; 1:1000 for WB), mouse anti-Aly/REF (Sigma-Aldrich, A9979; 1:1000 for WB), rabbit anti-NXF1 (ABclonal, A3488; 1:1000 for WB), mouse anti-TAP/NXF1 (Sigma-Aldrich, T1076; 1:1000 for WB), rabbit anti-THOC3 (ABclonal, A13230; 1:1000 for WB), rabbit anti-THOC6 (ABclonal, A17766; 1:1000 for WB), rabbit anti-NUP358 (ABclonal, A18596; 1:1000 for WB), rabbit anti-NUP98 (ABclonal, A0530; 1:1000 for WB), mouse anti-nuclear pore complex proteins (Abcam, Cambrige, UK, MAB414; 1:1000 for WB), rabbit anti-UAP56 (ABclonal, A8356; 1:1000 for WB), rabbit anti-β-actin (Abmart, Shanghai, China, P30002; 1:5000 for WB), mouse anti-His-Tag (ABclonal, AE003; 1:2000 for WB), mouse anti-GST-Tag (ABclonal, AE001; 1:2000 for WB), mouse anti-GAPDH (ABclonal, AC002; 1:5000 for WB), and mouse anti-α-tubulin (ABclonal, AC012; 1:5000 for WB). Horseradish peroxidase (HRP)-conjugated secondary antibodies: goat anti-mouse (Abmart, M21001) and goat anti-rabbit (Abmart, M21002) were used at 1:10,000 dilution in Western blot analysis. Alexa Fluor-conjugated secondary antibodies (488 and 546) were from Invitrogen (A11001, A11008, A11003, A11010).

### 2.3. Cell Culture

HEK293T and Vero cells from the ATCC were cultured in Dulbecco’s modified Eagle’s medium (DMEM; Gibco, Grand Island, NY, USA) supplemented with 10% (*v*/*v*) fetal bovine serum (FBS; Sigma-Aldrich) and 1% penicillin-streptomycin (Gibco) at 37 °C in 5% CO_2_. All cell lines were routinely tested for mycoplasma contamination.

### 2.4. Plasmids and Site-Directed Mutagenesis

Codon-optimized cDNA sequences encoding Nsp1 from SARS-CoV, SARS-CoV-2, MERS-CoV, HCoV-HKU1, HCoV-OC43, HCoV-NL63, and HCoV-229E were synthesized and cloned into the pCAGGS expression vector containing an N-terminal 3×Flag tag. Mutant Nsp1 protein expression plasmids were generated using the QuikChange II Site-Directed Mutagenesis Kit (Agilent, Santa Clara, CA, USA) and verified by Sanger sequencing. For protein purification in Escherichia coli, the human NXF1 was cloned into the pGEX-6P-1 plasmid with an N-terminal GST tag and the coronavirus Nsp1 was cloned into the pET28a plasmid with an N-terminal 6×His-tag.

### 2.5. Plasmid Transfection

Cells were transfected using Lipofectamine 3000, jetOPTIMUS or PEI (Yeasen Biotechnology, Shanghai, China, 40816ES10) according to the manufacturer’s protocols. For jetOPTIMUS transfection, 1 μg plasmid DNA was diluted in 100 μL jetOPTIMUS buffer, mixed with 1 μL jetOPTIMUS reagent, incubated for 10 min at room temperature, and added to cells. For Lipofectamine 3000, 1 μg plasmid DNA was mixed with 2 μL P3000 reagent in 50 μL Opti-MEM, and 1 μL Lipofectamine 3000 was diluted in 50 μL Opti-MEM; the two solutions were mixed thoroughly, incubated for 15 min, and added to cells.

### 2.6. RNA Fluorescence In Situ Hybridization (RNA-FISH) and Immunofluorescence (IF)

Cells grown on coverslips were fixed with 4% paraformaldehyde in PBS overnight at 4 °C for FISH. For immunofluorescence only, fixation was performed for 15 min at room temperature. After fixation, cells were permeabilized with 0.5% Triton X-100 (Sigma-Aldrich, T9284) in PBS for 15 min and washed twice with PBS. For RNA-FISH, cells were incubated with hybridization buffer containing 125 nM Oligo(dT)-CY5 probe at 37 °C for 3.5 h, followed by washes with FISH wash buffer (2× SSC, 10% formamide) and 0.5× SSC. For combined RNA-FISH and immunofluorescence, cells were blocked with 5% BSA in PBS for 30 min, incubated with primary antibodies at room temperature for 1 h, washed with PBS, and incubated with Alexa Fluor-conjugated secondary antibodies for 1 h. Nuclei were stained with Hoechst 33258 (1:10,000) for 5 min. Coverslips were mounted with ProLong Gold Antifade Reagent (Thermo Fisher Scientific). Images were acquired using an Olympus confocal microscope and processed with ImageJ and ImarisViewer software (v9.9.0).

### 2.7. Nuclear-Cytoplasmic Fractionation

Cells from one 10 cm dish were harvested and resuspended in 300 μL Fractionation Buffer A (15 mM Tris-HCl pH 7.5, 15 mM NaCl, 60 mM KCl, 1 mM EDTA, 0.5 mM EGTA, 0.5 mM spermidine, 10 U/mL Rnasin, 1× protease inhibitor cocktail). An equal volume of 2× lysis buffer (Fractionation Buffer A with 0.4% NP-40) was added, mixed by inversion, and incubated on ice for 5 min. The lysate was centrifuged at 400× *g* for 5 min. The supernatant was collected as the cytoplasmic fraction and clarified by centrifugation at 16,000× *g* for 3 min. The nuclear pellet was washed with 1 mL RLN buffer (50 mM Tris-HCl pH 7.5, 140 mM NaCl, 1.5 mM MgCl_2_, 10 mM EDTA, 10 U/mL RNasin, 0.25% NP-40) and collected as the nuclear fraction. Fractionation efficiency was validated by Western blot (GAPDH for cytoplasm, Histone H3 for nucleus) and qPCR (NEAT1 for nucleus, GAPDH for cytoplasm).

### 2.8. Immunoprecipitation (Co-IP)

Cells were lysed in cell lysis buffer (50 mM Tris-HCl pH 7.5, 150 mM NaCl, 1% IGEPAL CA-630, 0.1 mM Na_3_VO_4_, 1 mM NaF, 1 mM DTT, 1 mM EDTA, 1 mM PMSF, 1× protease inhibitor cocktail, 10% glycerol) for 30 min on ice with vortexing every 5 min. Lysates were sonicated briefly and clarified by centrifugation at 12,000× *g* for 5 min. For Flag IP, supernatants were incubated with Anti-Flag M2 Magnetic Beads at 4 °C for 4 h or overnight. Beads were washed five times with lysis buffer and proteins eluted with 200 μg/mL 3×Flag peptide. For endogenous IP, supernatants were incubated with specific antibodies or control IgG and Protein A/G agarose at 4 °C for 4 h or overnight. Beads were washed five times, and proteins were eluted with 2× SDS sample buffer. Where indicated, RNase A (10 μg/mL) or RNasin (40 U/mL) was added during lysis.

### 2.9. Recombinant Protein Expression and GST Pull-Down Assays

Recombinant GST, GST-NXF1, and His-tagged Nsp1 constructs were expressed in E. coli BL21(DE3) induced with 0.2 mM IPTG at 16 °C for 16 h. Bacterial pellets were lysed by sonication and purified using Glutathione Sepharose 4B or Ni-NTA agarose resins. For pull-down assays, 5 μg of immobilized GST or GST-NXF1 was incubated with 2 μg of purified His-Nsp1 in pull-down buffer (20 mM Tris-HCl pH 7.5, 150 mM NaCl, 1 mM DTT, 0.5% NP-40, 1× protease inhibitor cocktail) at 4 °C for 2 h. Beads were washed five times, eluted in 2× SDS sample buffer, and analyzed by Western blot using anti-His and anti-GST antibodies.

### 2.10. Proximity Ligation Assay (PLA)

PLA was performed using the Duolink In Situ Red Starter Kit (Sigma-Aldrich) according to the manufacturer’s instructions. Cells on coverslips were fixed with 4% paraformaldehyde for 15 min and permeabilized with 0.5% Triton X-100 for 15 min. After blocking with Duolink blocking solution at 37 °C for 60 min, cells were incubated with primary antibodies (anti-Flag and anti-NXF1) diluted in Duolink antibody diluent at 37 °C for 60 min. PLA probes (PLUS and MINUS) were added and incubated at 37 °C for 60 min. Ligation and amplification were performed according to the manufacturer’s protocol. Subsequently, cells were incubated with primary antibodies (anti-Flag and anti-NXF1) at 4 °C overnight, followed by incubation with Alexa Fluor-conjugated secondary antibodies for 1 h. Nuclei were stained with Hoechst 33258. Coverslips were mounted and images acquired using confocal microscopy.

### 2.11. Structural Modeling

Structural models of hCoV Nsp1 proteins in complex with human NXF1-RRM (PDB: 1K3X) were generated using AlphaFold3. The structures of SARS-CoV-2, MERS-CoV, HCoV-229E, HCoV-HKU1, HCoV-NL63, and HCoV-OC43 were superposed to the SARS-CoV structure using Coot, yielding R.M.S.D. values of 1.1 Å, 3.5 Å, 2.5 Å, 2.1 Å, 2.4 Å, and 2.6 Å, respectively. The electrostatic surface potential maps of the predicted α-helices that bind NSP1 in these structures were generated using the Vacuum electrostatics program in PyMOL (v4.6.0).

### 2.12. Phylogenetic Analysis

Representative coronavirus Nsp1 protein sequences were obtained from the NCBI Protein database (https://www.ncbi.nlm.nih.gov/protein/, accessed on 20 March 2026), including seven coronavirus species associated with human infection (Supplementary Table S1). A total of 32 Nsp1 amino acid sequences were used for phylogenetic analysis. We then extracted Nsp1 sequences from the corresponding ORF1a protein sequences according to the annotated Nsp1 features and amino acid coordinates in the NCBI protein database.

Multiple sequence alignment was performed using MAFFT v7.526 with the L-INS-i strategy (--localpair --maxiterate 1000). Poorly aligned regions were removed using trimAl v1.5.rev1 with the automated1 trimming strategy. A maximum-likelihood phylogenetic tree was constructed using IQ-TREE v2.2.2.7. The optimal amino acid substitution model was selected using ModelFinder based on the Bayesian Information Criterion (BIC), and the WAG model with a discrete Gamma distribution (WAG + G4) was identified as the best-fitting model. Branch support was evaluated using 1000 ultrafast bootstrap replicates and 1000 SH-aLRT replicates. Phylogenetic trees were visualized using R v4.5.0 with the ape v5.8.1 and ggtree v4.0.5 packages.

### 2.13. Statistical Analysis

All data are presented as mean ± SD from at least three independent experiments. Statistical analyses were performed using GraphPad Prism 8.0.2 and Microsoft Office Excel. Comparisons between two groups were analyzed using unpaired two-tailed Student’s *t*-test. *p* < 0.05 was considered statistically significant (* *p* < 0.05, ** *p* < 0.01, *** *p* < 0.001, **** *p* < 0.0001). Fluorescence and Western blot images were quantified using ImageJ 1.54f. Co-localization analysis was performed using ImageJ 1.54f, and three dimensional reconstruction and statistical analysis of fluorescence signals were carried out using ImarisViewer. Enrichment analyses were performed using DAVID (https://davidbioinformatics.nih.gov/home.jsp, accessed on 20 March 2026), and bioinformatics figures were generated using R 4.2.2. All sample sizes and statistical tests are indicated in the corresponding figure legends.

## 3. Results

### 3.1. Human Coronavirus NSP1 Proteins Differentially Inhibit Poly(A) RNA Nuclear Export

**Figure 1:**
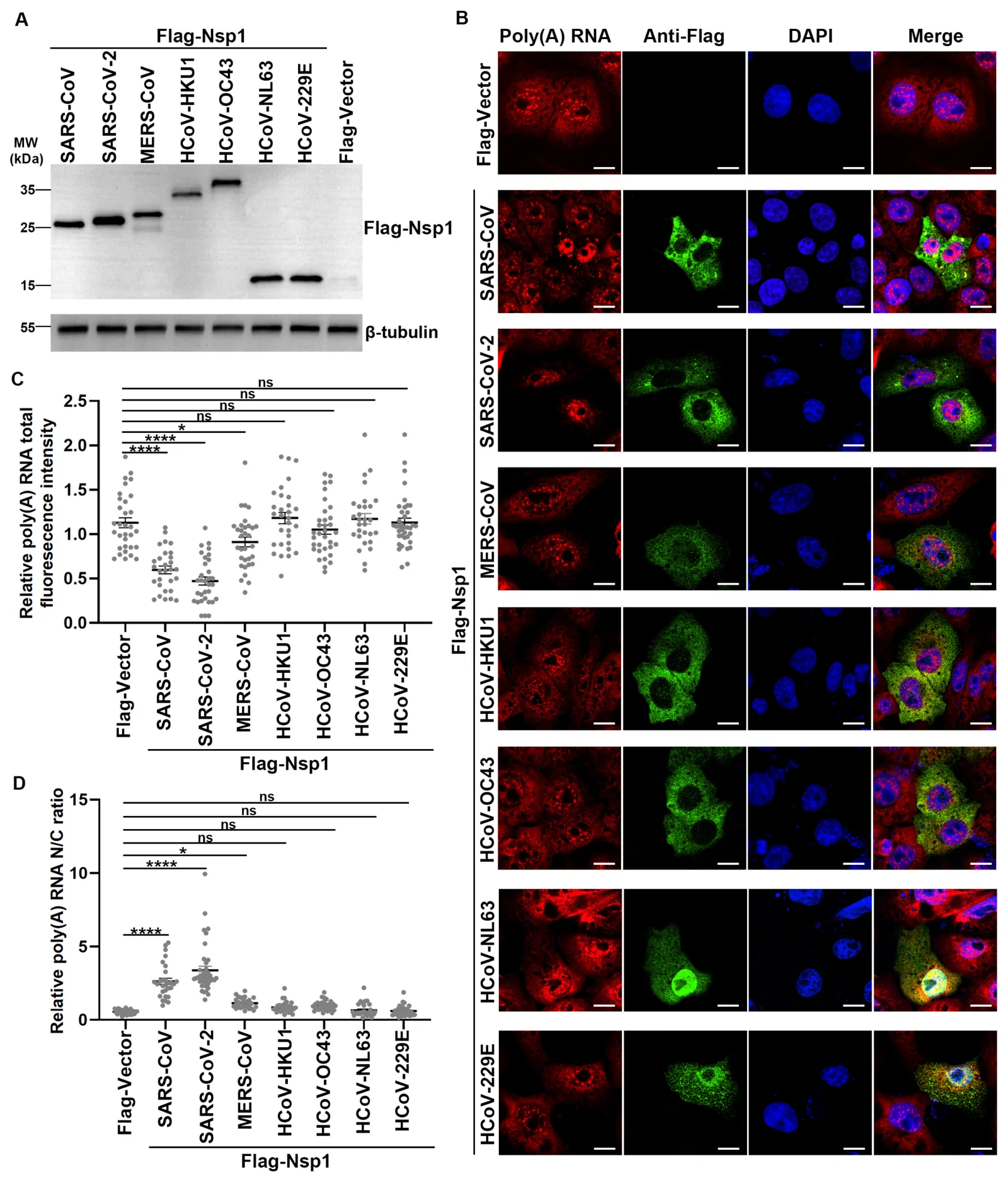
Human coronavirus Nsp1 suppresses host cellular mRNA nuclear export. (**A**) Vero cells were transfected with a Flag-tagged empty vector or Flag-Nsp1 derived from the indicated human coronaviruses (SARS-CoV, SARS-CoV-2, MERS-CoV, HCoV-HKU1, HCoV-OC43, HCoV-229E, and HCoV-NL63) for 24 h. Cell lysates were then collected and subjected to Western blot analysis using an anti-Flag antibody. β-Tubulin was used as a loading control. (**B**) Representative RNA fluorescence in situ hybridization (RNA-FISH) images showing the intracellular localization of poly(A) RNA(red) in Vero cells transfected with Flag-tagged empty vector or Flag-tagged Nsp1(green) from the indicated coronaviruses for 24 h. Poly(A) RNA was visualized using a Cy3-labeled oligo(dT) probe. Transfected cells were detected by staining with an anti-Flag antibody, and nuclei were counterstained with DAPI(blue). Scale bars, 10 μm. (**C**) Quantification of total cellular poly(A) RNA signal intensity per cell in control and Flag-Nsp1-expressing cells. (**D**) Quantitative analysis of the nuclear-to-cytoplasmic (N/C) intensity ratio of poly(A) RNA across the indicated groups. Data is presented as mean ± SD from three independent experiments (*n* = 30 cells per group). *, *p* < 0.05; ****, *p* < 0.0001; ns, not significant. Data in (**A**–**D**) are representative of 3 independent experiments.

To determine whether the blockade of host mRNA nuclear export is a shared functional hallmark across human coronaviruses, we cloned cDNA sequences encoding Nsp1 from seven hCoV strains representing both Alphacoronavirus (HCoV-229E, HCoV-NL63) and Betacoronavirus (Lineage A: HCoV-HKU1, HCoV-OC43; Lineage B: SARS-CoV, SARS-CoV-2; Lineage C: MERS-CoV) genera into mammalian expression vectors containing N-terminal Flag tags. Western blot analysis of transfected Vero cell lysates confirmed robust expression of all seven Flag-NSP1 constructs at their expected molecular weights (Figure 1A). To evaluate host transcript distribution, we performed RNA fluorescence in situ hybridization (RNA-FISH) using Cy3-labeled oligo(dT) probes to track endogenous poly(A) RNA localization in transfected Vero cells. The results showed that expression of Flag-tagged Nsp1 from SARS-CoV, SARS-CoV-2, and MERS-CoV resulted in significant nuclear retention of poly(A) RNA, characterized by strong nuclear accumulation and reduced cytoplasmic staining. In contrast, Nsp1 from HCoV-HKU1, HCoV-OC43, HCoV-NL63, and HCoV-229E did not induce appreciable nuclear accumulation of poly(A) RNA, whose distribution remained comparable to that observed in control cells. (Figure 1B). Quantification of cellular fluorescence revealed that total poly(A) RNA signal intensity was significantly reduced in cells expressing SARS-CoV or SARS-CoV-2 Nsp1, with a more modest reduction observed in cells expressing MERS-CoV Nsp1, whereas the other Nsp1 constructs showed little change compared to empty-vector controls (Figure 1C). However, quantitative measurement of the nuclear-to-cytoplasmic (N/C) intensity ratio demonstrated statistically significant increases only in cells expressing Nsp1 from SARS-CoV, SARS-CoV-2, and MERS-CoV, but not in cells expressing Nsp1 from HCoV-HKU1, HCoV-OC43, HCoV-NL63, or HCoV-229E, compared with the corresponding controls (Figure 1D). Together, these findings demonstrate differential inhibition of host poly(A) RNA nuclear export by hCoV Nsp1 proteins, with this activity observed for Nsp1 proteins from SARS-CoV, SARS-CoV-2, and MERS-CoV, but not for those from the endemic seasonal hCoVs tested.

### 3.2. Nsp1 Interaction with NXF1 Correlates with Inhibition of Host mRNA Nuclear Export

**Figure 2:**
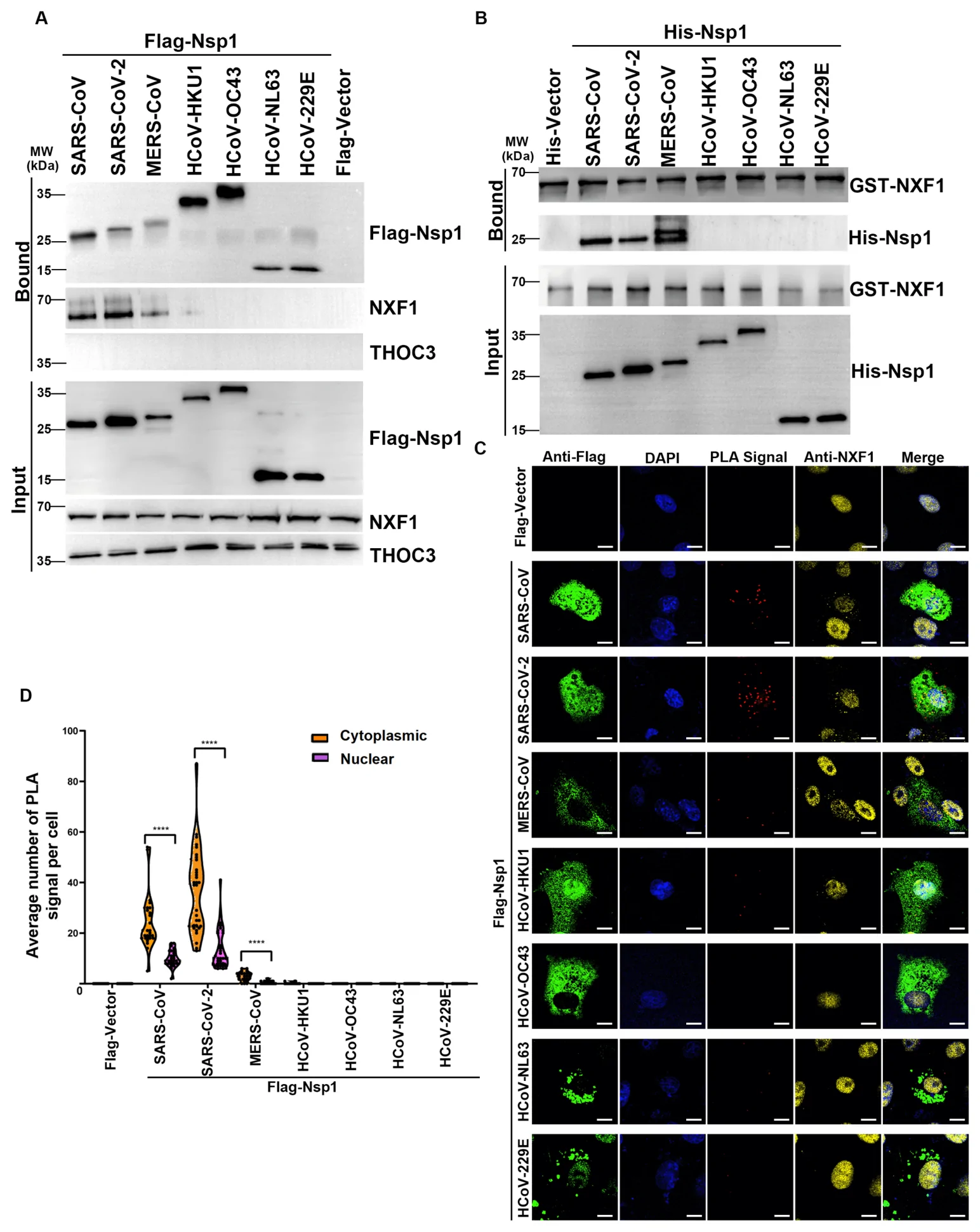
Nsp1 directly recruits the host mRNA nuclear export receptor NXF1. (**A**) Co-immunoprecipitation (Co-IP) analysis of the interaction between Nsp1 and host mRNA export factors. HEK293T cells were transfected with Flag-vector or Flag-Nsp1 from indicated coronaviruses for 24 h. Cell lysates were immunoprecipitated with anti-Flag M2 magnetic beads and immunoblotted with antibodies against NXF1, THOC3, and Flag. (**B**) The in vitro GST pull-down assay was performed using recombinant GST-NXF1 and His-tagged Nsp1 proteins from the indicated coronaviruses, purified from *E. coli*. Pull-down and input proteins were analyzed by Western blotting using anti-His and anti-GST antibodies. (**C**) Vero cells were transfected with Flag-tagged empty vector or Flag-Nsp1 from the indicated coronaviruses for 24 h. The interaction between Nsp1(green) and NXF1(yellow) was detected by an in situ proximity ligation assay (PLA). Red puncta indicate PLA interaction signals. Nuclei were counterstained with DAPI(blue). Scale bars, 10 μm. (**D**) Quantification of PLA puncta per cell in nuclear and cytoplasmic fractions. Bars represent mean ± SD of three independent experiments. ****, *p* < 0.0001. Data in (**A**–**D**) are representative of 3 independent experiments.

SARS-CoV-2 Nsp1 has been reported to interact with the cellular mRNA export receptor NXF1 [28,29]. To determine whether NXF1 interaction differs among hCoV Nsp1 proteins and correlates with their ability to inhibit host mRNA nuclear export, we performed co-immunoprecipitation (Co-IP) assays in HEK293T cells transfected with Flag-tagged Nsp1 constructs from the seven hCoVs. Cell lysates were immunoprecipitated using anti-Flag M2 magnetic beads and probed for endogenous export factors. Immunoblotting revealed that Nsp1 from SARS-CoV, SARS-CoV-2, and MERS-CoV robustly co-precipitated endogenous NXF1, whereas the endemic hCoV Nsp1 proteins showed little to no detectable NXF1 co-precipitation. In contrast, THOC3, an essential component of the upstream THO/TREX complex, was not detected in the Flag-Nsp1 immunoprecipitants (Figure 2A).

To determine whether Nsp1 directly interacts with NXF1 in the absence of host bridging factors or RNA, we purified recombinant His-tagged Nsp1 variants and GST-tagged human NXF1 from E. coli and conducted in vitro GST pull-down assays. GST-NXF1 efficiently pulled down His-tagged Nsp1 from SARS-CoV, SARS-CoV-2, and MERS-CoV, whereas no robust binding was detected for HCoV-HKU1, HCoV-OC43, HCoV-NL63, or HCoV-229E; GST control protein showed no detectable binding (Figure 2B).

To confirm the Nsp1-NXF1 interaction in a cellular context, we performed in situ proximity ligation assays (PLA) in Vero cells expressing Flag-tagged Nsp1 constructs. Fluorescent PLA puncta representing Nsp1-NXF1 interactions were observed in cells expressing Nsp1 from SARS-CoV, SARS-CoV-2, and MERS-CoV, whereas control vector cells and cells expressing endemic hCoV Nsp1 displayed virtually no PLA signal (Figure 2C). Compartmental quantification of PLA puncta showed abundant interaction signals distributed within both nuclear and cytoplasmic compartments for SARS-CoV, SARS-CoV-2, and MERS-CoV, but not for the endemic hCoV NSP1 proteins (Figure 2D).

Together, these results demonstrate that Nsp1 proteins from SARS-CoV, SARS-CoV-2, and MERS-CoV directly interact with NXF1, whereas Nsp1 proteins from the endemic seasonal hCoVs tested did not show detectable NXF1 interaction, consistent with their limited ability to inhibit host mRNA nuclear export.

### 3.3. The N-Terminal Acidic Patch Determines Nsp1-Mediated Inhibition of Poly(A) RNA Nuclear Export

**Figure 3:**
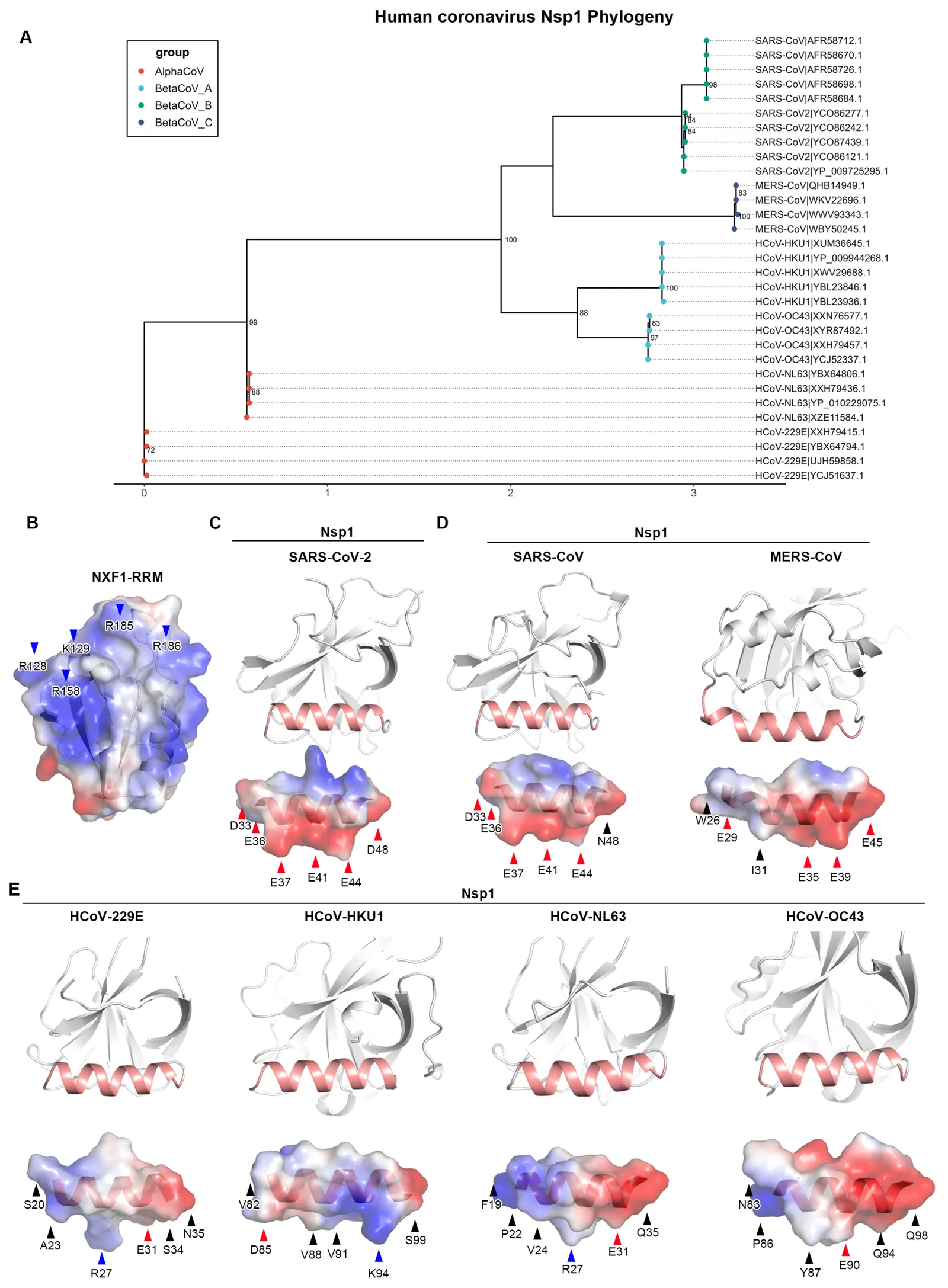
Phylogenetic and structural analysis of the N-terminal acidic patch in hCoV Nsp1 proteins. (**A**) Phylogenetic tree of Nsp1 proteins from seven representative human coronaviruses (hCoVs), including Alphacoronavirus (HCoV-229E, HCoV-NL63) and Betacoronavirus lineages A (HCoV-HKU1, HCoV-OC43), B (SARS-CoV, SARS-CoV-2), and C (MERS-CoV). (**B**) Structural modeling and electrostatic surface potential of the NXF1 RNA recognition motif (NXF1-RRM). Positively charged residues on NXF1 (R128, K129, R158, R185, and R186; blue arrowheads) are indicated. (**C**) Structural modeling and electrostatic surface potential of SARS-CoV-2 Nsp1. Negatively charged acidic residues (D33, E36, E37, E41,E44 and D48; red arrowheads). (**D**) Structural models and electrostatic surface potentials of Nsp1 from highly pathogenic human coronaviruses (SARS-CoV and MERS-CoV). Key residues within the corresponding acidic region are highlighted with red arrowheads, and non-conserved amino acid residues in this region are indicated with black arrowheads. (**E**) Structural models and electrostatic surface potentials of Nsp1 from endemic human coronaviruses (HCoV-229E, HCoV-HKU1, HCoV-NL63, and HCoV-OC43).

To analyze the evolutionary conservation of Nsp1, we built a phylogenetic tree of Nsp1 proteins from representative human coronaviruses, classifying them into distinct groups corresponding to Alpha- and Betacoronavirus genera. As expected, the Nsp1 proteins from Alphacoronavirus and Betacoronavirus lineages were evolutionarily distinct from one another (Figure 3A). A previous study identified a surface-exposed acidic patch comprising D33, E36, E37, and E41 that mediates interaction with the NXF1 RRM domain; charge-reversal mutations at these residues disrupted the Nsp1-NXF1 interaction and restored mRNA nuclear export [28]. Consistent with this mechanism, our RNA-FISH and interaction assays (Figure 1D and Figure 2A–D) showed that significant NXF1 binding and mRNA export inhibition were detected for SARS-CoV, SARS-CoV-2, and MERS-CoV Nsp1, but not for Nsp1 from the endemic hCoVs.

Structural computational modeling was then performed to inspect the interaction surface with the NXF1 RNA recognition motif (NXF1-RRM), which features a prominent basic patch (R128, K129, R158, R185, R186) (Figure 3B). In line with this basic surface, SARS-CoV-2 Nsp1 proteins possess a negatively charged acidic patch in their N-terminal domains, composed of six acidic residues (D33, E36, E37, E41, E44, and D48) (Figure 3C). SARS-CoV and MERS-CoV Nsp1 retain a similar acidic region, although the residue corresponding to D48 in SARS-CoV Nsp1 is replaced by N48, whereas the residues corresponding to D33 and E37 in MERS-CoV Nsp1 are replaced by W26 and I31, respectively, resulting in partial loss of acidic residues within the region (Figure 3D). In contrast, Nsp1 proteins from HCoV-229E, HCoV-HKU1, HCoV-NL63, and HCoV-OC43 lack five acidic residues corresponding to the acidic patch identified in SARS-CoV-2 Nsp1. Specifically, in HCoV-229E Nsp1, the residues corresponding to D33, E36, E37, E44, and D48 are replaced by S20, A23, R27, S34, and N35, respectively. In HCoV-HKU1 Nsp1, the corresponding residues D33, E37, E41, E44, and D48 are replaced by V82, V88, V91, K94, and S99, respectively. In HCoV-NL63 Nsp1, D33, E36, E37, E41, and D48 are replaced by F19, P22, V24, R27, and Q35, respectively. Similarly, in HCoV-OC43 Nsp1, D33, E36, E37, E44, and D48 are replaced by N83, P86, Y87, Q94, and Q98, respectively. The progressive loss of these acidic residues correlates with reduced NXF1 interaction and impaired inhibition of host mRNA nuclear export (Figure 3E).

Together, these findings identify the conserved N-terminal acidic patch as a key determinant of Nsp1-NXF1 interaction and Nsp1-mediated inhibition of host mRNA nuclear export.

### 3.4. Nsp1 Inhibits Poly(A) RNA Nuclear Export by Disrupting NXF1 Interactions with the mRNA Export Machinery

**Figure 4:**
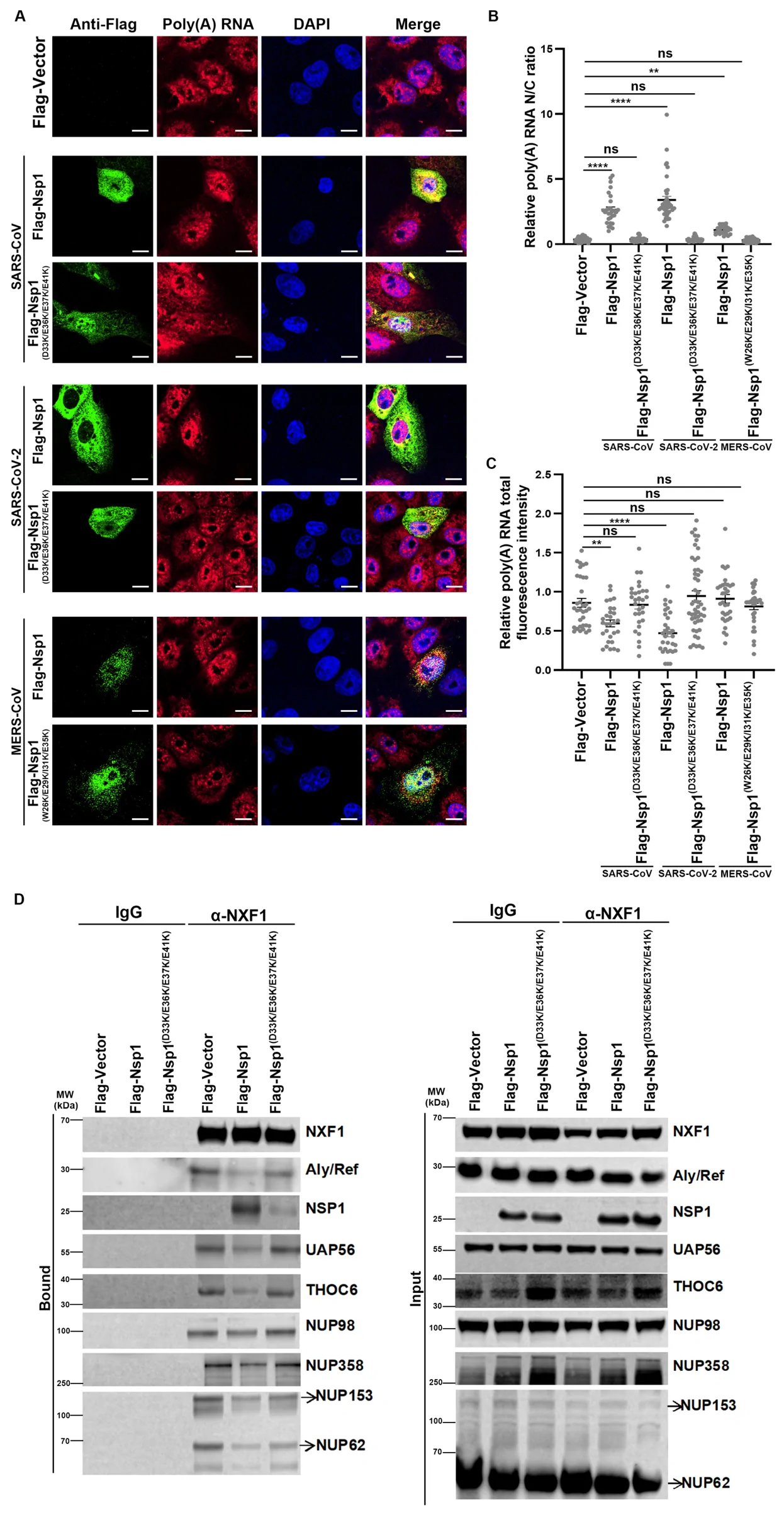
Mutations at the Nsp1 binding interface abolish NXF1 interaction and restore cellular mRNA export. (**A**) Vero cells were transfected with Flag-tagged empty vector, wild-type Flag-Nsp1, or the corresponding quadruple mutant Flag-Nsp1^(D33K/E36K/E37K/E41K)^ from SARS-CoV, SARS-CoV-2, or Flag-Nsp1^(W26K/E29K/I31K/E35K)^ MERS-CoV for 24 h. The subcellular distribution of poly(A) RNA was then evaluated by RNA-FISH. Poly(A) RNA (Cy3, red), Flag-tagged Nsp1 (green), and nuclei (DAPI, blue) were visualized by confocal microscopy. Scale bars, 10 μm. (**B**) Quantitative ratio of nuclear-to-cytoplasmic (N/C) poly(A) RNA signals. Data represent mean ± SD from three independent replicates. (**C**) Quantification of total cellular poly(A) RNA fluorescence intensity in cells. **, *p* < 0.01; ****, *p* < 0.0001; ns, not significant. Data represent mean ± SD from three independent replicates. (**D**) Endogenous co-immunoprecipitation in HEK293T cells transfected with Flag-Nsp1 or Flag-Nsp1^(D33K/E36K/E37K/E41K)^ from SARS-CoV-2 for 24 h. Cell lysates were subjected to IP with anti-NXF1 antibody or IgG control and analyzed by Western blot using antibodies against TREX components (Aly/Ref, UAP56, THOC6) and nuclear pore complex proteins (NUP98, NUP358, NUP153, NUP62). Arrows indicate the specific target bands of NUP153 and NUP62. Data in (**A**–**D**) are representative of 3 independent experiments.

To test whether this modeled electrostatic patch is functionally required for Nsp1-mediated inhibition of mRNA nuclear export, we selected four of the six acidic residues within the patch (D33, E36, E37, and E41) based on their previously reported functional importance in Nsp1-NXF1 interaction [28]. We generated a charge-reversal mutant in which these four residues were replaced with basic lysine residues (Flag-Nsp1-D33K/E36K/E37K/E41K for SARS-CoV and SARS-CoV-2, Nsp1-W26K/E29K/I31K/E35K for MERS-CoV, designated as Flag-Nsp1 mutant). We evaluated the effect of wild-type Nsp1 (WT) versus the quadruple mutant (mut) on poly(A) RNA localization using RNA-FISH in Vero cells. Expression of WT Nsp1 from SARS-CoV, SARS-CoV-2, or MERS-CoV induced marked nuclear accumulation of poly(A) RNA, whereas the Nsp1-Mut constructs completely lost their ability to inhibit mRNA nuclear export, resulting in a distribution pattern indistinguishable from that of empty-vector controls (Figure 4A). Quantitative analysis confirmed that the N/C ratio was significantly elevated in WT Nsp1-expressing cells but returned to baseline in Nsp1-Mut-expressing cells (Figure 4B), whereas total cellular poly(A) RNA intensity remained largely unchanged relative to the control group, with the exception of cells expressing WT Nsp1 from SARS-CoV and SARS-CoV-2 (Figure 4C).

To determine whether loss of export suppression in Nsp1-Mut was due to loss of binding to NXF1 and whether WT Nsp1 disrupts downstream NPC associations, we performed co-immunoprecipitation experiments in HEK293T cells expressing WT or Mut SARS-CoV-2 Nsp1 using an anti-NXF1 antibody or an IgG control. Immunoblot analysis showed that WT Nsp1 co-precipitated with endogenous NXF1, whereas the Nsp1-Mut protein completely lost its ability to interact with NXF1. Furthermore, endogenous NXF1 immunoprecipitates from cells expressing vector or Nsp1-Mut retained interactions with mRNP export adaptors (Aly/REF, UAP56, THOC6) and key NPC nucleoporins (NUP98, NUP358, NUP153, NUP62). In contrast, WT Nsp1 expression markedly disrupted the interactions between NXF1 and these mRNA export factors and NPC nucleoporins (Figure 4D).

These findings demonstrate that Nsp1 targets NXF1 through its N-terminal acidic patch to disrupt the mRNA export machinery and inhibit host mRNA nuclear export.

## 4. Discussion

Host cell shutoff is a common viral strategy to prioritize viral protein synthesis and suppress host innate immune responses [22,30,31]. While previous work has highlighted the role of SARS-CoV-2 Nsp1 in translational repression via ribosome cleft plugging [11,12], the scope, evolutionary conservation, and structural mechanics of Nsp1-mediated nuclear export inhibition remained incompletely characterized. In this study, we demonstrate that Nsp1 from the highly pathogenic hCoVs SARS-CoV, SARS-CoV-2, and MERS-CoV blocks host mRNA nuclear export by directly targeting the core export receptor NXF1 through a conserved N-terminal electrostatic interface. In contrast, Nsp1 from the endemic hCoVs HCoV-HKU1, HCoV-OC43, HCoV-NL63, and HCoV-229E did not significantly inhibit mRNA export and showed no detectable interaction with NXF1.

Our comparative analysis across seven human coronaviruses demonstrates that nuclear export suppression is not a conserved feature of all coronavirus Nsp1 proteins. Robust inhibition was observed for Nsp1 from SARS-CoV, SARS-CoV-2, and MERS-CoV, whereas Nsp1 from the endemic seasonal hCoVs HCoV-229E, HCoV-NL63, HCoV-HKU1, and HCoV-OC43 showed little effect on mRNA export. These findings reveal functional differences in Nsp1-mediated host shutoff among human coronaviruses [31,32]. Crucially, single-cell quantification showed that total poly(A) RNA fluorescence was reduced by SARS-CoV and SARS-CoV-2 Nsp1 and modestly decreased by MERS-CoV Nsp1. Nevertheless, the pronounced nuclear accumulation of poly(A) RNA indicates that inhibition of nuclear export is a major consequence of Nsp1 expression, rather than simply reflecting global loss of cellular poly(A) RNA [30].

At the molecular level, we identified NXF1 as the direct functional target of highly pathogenic hCoV Nsp1. NXF1, in complex with NXT1, serves as the primary receptor that transports mature mRNPs across the nuclear pore complex (NPC) by interacting with FG-repeat nucleoporins [33,34]. By directly binding NXF1, Nsp1 targets a central bottleneck in host mRNA export. Co-IP and GST pull-down assays confirmed a direct, RNA-independent interaction between NXF1 and Nsp1 from SARS-CoV, SARS-CoV-2, and MERS-CoV, while PLAs verified these interactions in intact cells [30]. In contrast, Nsp1 proteins from the endemic hCoVs showed little or no detectable NXF1 binding or PLA signals.

Structural modeling revealed that this interaction is driven by electrostatic complementarity. A conserved acidic patch in the N-terminal region of SARS-CoV-2 Nsp1, comprising D33, E36, E37, and E41, engages the positively charged RNA recognition motif (RRM) of NXF1. Charge-reversal mutations (D33K/E36K/E37K/E41K) completely abolished the Nsp1-NXF1 interaction and fully restored host poly(A) RNA nuclear export. Furthermore, analysis of endogenous NXF1 complexes demonstrated that Nsp1 binding disrupts the interaction between NXF1 and mRNP export adaptors, such as Aly/REF and UAP56, as well as key NPC nucleoporins, thereby inhibiting host mRNA nuclear export.

This mechanism significantly expands our understanding of the multi-layered strategies used by coronaviruses to counteract host antiviral responses [29,30]. Coronaviruses target multiple levels of host gene expression and RNA metabolism, including ribosomal translation through the Nsp1 C-terminus [11,12], nucleocytoplasmic transport through the Nsp1 N-terminus and ORF6 [26,29], and nuclear pore function through Nsp9 binding to Nup62 [35]. Together, these mechanisms provide complementary routes for suppressing host gene expression and antiviral responses.

In summary, our study defines a conserved electrostatic interface between Nsp1 from highly pathogenic human coronaviruses and host NXF1 that drives host mRNA nuclear retention. The conservation of this interface across SARS-CoV, SARS-CoV-2, and MERS-CoV highlights Nsp1-NXF1 interaction as a potential target for broad-spectrum antiviral strategies aimed at restoring host mRNA export and innate immune surveillance.

## Data Availability

All data generated or analyzed during this study are included in this article and its Supplementary Information.

## Supplementary Materials

The following supporting information can be downloaded at: https://www.mdpi.com/article/10.3390/v18101157/s1, Table S1: Information on the Nsp1 proteins used in this study.
