# Supplementary material for: An N-Terminal Acidic Patch Underlies Differential Inhibition of mRNA Nuclear Export by Human Coronavirus Nsp1 Proteins
Source: Viruses. 2026 Oct 18;18(10):1157. doi: 10.3390/v18101157 (PMC13709700; doi:10.3390/v18101157)
Supplement: Supplementary file 1 [file viruses-18-01157-s001.zip › viruses-4617483-supplementary.pdf]

Table S1. Information on the Nsp1 proteins used in this study

Viruses	GenBank Number	Amino Acid Sequence
SARS-CoV	NP_828860.2	MESLVLGVNEKTHVQLSLPVLQVRDVLVRGFGDSVEEALSEAREHLK<br>NGTCGLVELEKGVLPQLEQPYVFIKRSDALSTNHGHKVVELVAEMDG<br>IQYGRSGITLGVLVPHVGETPIAYRNVLLRKNGNKGAGGHSYGIDLKS<br>YDLGDELGTDPIEDYEQNWNTKHGSGALRELTRELNGG
SARS-CoV-2	WFQ19818.1	MESLVPGFNEKTHVQLSLPVLQVRDVLVRGFGDSVEEVLSEARQHLK<br>DGTCGLVEVEKGVLPQLEQPYVFIKRSDARTAPHGHVMVELVAELEGI<br>QYGRSGETLGVLVPHVGEIPVAYRKVLLRKNGNKGAGGHSYGADLKS<br>FDLGDELGTDPYEDFQENWNTKHSSGVTRELMRELNGG
MERS-CoV	AGV08464.1	MSFVAGVTAQGARGTYRAALNSEKHQDHVSLTVPLCGSGNLVEKLSP<br>WFMDGENAYEVVKAMLLKKEPLLYVPIRLAGHTRHLPGPRVYLVERL<br>IACENPFMVNQLAYSSSANGSLVGTTLQGKPIGMFFPYDIELVTGKQNI<br>LLRKYGRGGYHYTPFHYERDNTSCPEWMDDFEADPKGKYAQNLLKK<br>L
HCoV-HKU1	ABD75583.1	MIKTSKYGLGFKWAPEFRWLLPDAAEELASPMKSDEGGLCPSTGQAM<br>ESVGFVYDNHVKIDCRCILGQEWHVQSNLIRDIFVHEDLHVVEVLTKT<br>AVKSGTAILIKSPLHSLGGFPKGYVMGLFRSYKTKRYVVHHLSMTTSTT<br>NFGEDFLGWIVPFGFMPSYVHKWFQFCRLYIEESDLIISNFKFDDYDFSV<br>EDAYAEVHAEPKGKYSQKAYALLRQYRGIKPVLFVDQYGCDYSGKLA<br>ACLQAYGHYSLQDMRQKQSVWLANCDFDIVVAWHVVRDSRFVMRL<br>QTIATICGIKYVAQPTEDVVDGDVVIREPVHLLSADAIVLKLPS
HCoV-OC43	XXH79328.1	MSKINKYGLELHWAPEFPWMFEDAEEKLDNPSSSEVDMICSTTAQKLE<br>TDGICPENHVMVDCRRLLKQECCVQSSLIREIVMNASPYYLEVLLQDA<br>LQSREAVLVTTPLGMSLEACYVRGCNPKGWTMGLFRRRSVCNTGRCT<br>VNKHVAYQLYMIDPAGVCLGAGQFVGWVIPLAFMPVQSRKFIVPWV<br>MYLRKRGEKGAYNKDHGCGGFGHVYDFKVEDAYDQVHDEPKGKFS<br>KKAYALIRGYRGVKPLLYVDQYGCDYTGSLADGLEAYADKTLQEMK<br>ALFPTWSQELPFDVIVAWHVVRDPRYVMRLQSAATICSVAYVANPTE<br>DLCDGSVVIKEPVHVYADDSIILRQYN
HCoV-NL63	XPR31527.1	MFYNQVTLAVASDSEISGFGFAIPSVAVRTYSEAAAQGFQACRFVAFG<br>LQDCVTGINDDDYVIALTGTNQLCAKILPFSDRPLNLRGWLIFSNSNY<br>VLQDFDVVFGHGAG
HCoV-229E	NP_835344.1	MACNRVTLAVASDSEISANGCSTIAQAVRRYSEAASNGFRACRFVSLD<br>LQDCIVGIADDTYVMGLHGNQTLFCNIMKFSDRPFMLHGWLVFSNSN<br>YLLEEFDVVFGKRGGG
